# Mirogabalin for Treatment of Neuropathic Pain and Associated Sleep Interference: An Updated Meta‐Analysis

**DOI:** 10.1002/ejp.70112

**Published:** 2025-09-19

**Authors:** Rafael Batista João, Jilly Octoria Tagore Chan, André Batista João, Luísa Mendes Araújo, Julyana Medeiros Dantas

**Affiliations:** ^1^ Neurology and Neurophysiology Department Goiânia Neurological Institute Goiânia Goiás Brazil; ^2^ Division of Neurology, Department of Internal Medicine São Vicente de Paulo Charity Hospital Jundiaí São Paulo Brazil; ^3^ Faculty of Medicine University of Indonesia Depok Wijaya Karya Indonesia; ^4^ Department of Anesthesiology José Alencar City Hospital São Bernardo do Campo São Paulo Brazil; ^5^ Department of Internal Medicine Federal University of Pará Belém Pará Brazil; ^6^ Department of Internal Medicine Federal University of Rio Grande Do Norte Natal Rio Grande do Norte Brazil

**Keywords:** Gabapentinoids, Mirogabalin, neuropathic pain, pain medicine, sleep

## Abstract

**Background and Objective:**

Neuropathic pain (NeP) is a common and debilitating consequence of several neurological conditions. Gabapentinoids are used on a large scale for the treatment of both central and peripheral NeP. Mirogabalin, a novel gabapentinoid, has been proposed as a promising treatment for this condition; however, its efficacy and safety profile still need to be determined in clinical practice. In this systematic review and meta‐analysis, we assessed the efficacy on pain intensity reduction, effects on sleep interference by pain, and safety of mirogabalin compared with placebo in patients affected by NeP.

**Databases and Data Treatment:**

We searched PubMed, Cochrane Library, Embase, Web of Science, and ClinicalTrials.gov databases for randomised controlled trials (RCTs) comparing mirogabalin with placebo in patients experiencing central or peripheral NeP. We computed mean differences (MD) and pooled risk ratios (RR) for continuous and binary outcomes, respectively, with 95% confidence intervals (CI). Pain was measured on a 0 to 10 numerical rating scale.

**Results:**

We included 6 RCTs involving 3048 patients. The mean age was 60.6 years, and 64.7% were male. When compared with placebo, patients treated with mirogabalin had a significant decrease in average daily pain (MD −0.60; 95% CI −0.75 to −0.45; *p* < 0.001) and in pain‐related sleep interference scores (MD −0.66; 95% CI −0.81 to −0.51; *p* < 0.001). The mirogabalin group showed a higher rate of substantial pain relief (≥ 50%) compared with the placebo group (RR 1.27; 95% CI 1.10 to 1.46; *p* = 0.001). Nonetheless, treatment with mirogabalin increased the risk of weight gain, peripheral oedema, somnolence and dizziness.

**Conclusion:**

In this meta‐analysis of RCTs evaluating patients with central and peripheral NeP, mirogabalin significantly improved pain and decreased sleep interference by pain, as compared with placebo; however, there was an increased risk of adverse events.

**Significance:**

This meta‐analysis refines the current understanding of mirogabalin by demonstrating modest yet consistent benefits in reducing pain and pain‐related sleep interference across neuropathic pain syndromes. The results contribute to ongoing efforts to optimise neuropathic pain management and provide more robust evidence to support clinical decision‐making and guideline development.

**Abbreviations**
CI: confidence interval
MD: mean difference
MGB: mirogabalin
NeP: neuropathic pain
NNT: number needed to treat
PRISMA: Preferred Reporting Items for Systematic Reviews and Meta‐Analyses
PROSPERO: International Prospective Register of Systematic Reviews
RCT: randomised controlled trial
RR: risk ratio
RoB2: risk of bias 2
SF‐MPQ: Short‐form McGill Pain Questionnaire
VGCC: voltage‐gated calcium channels

## 1 Introduction

Neuropathic pain (NeP) is a condition that affects up to 7%–10% of the general population (Bouhassira 2019; Bouhassira et al. 2008; van Hecke et al. 2014), impairs quality of life, and is associated with considerable public health costs (O'Connor 2009). Several common neurological conditions are related to NeP, including diabetic peripheral neuropathy (Jensen et al. 2021), post‐herpetic neuralgia (Tang et al. 2023), spinal cord injury (Widerström‐Noga 2023), and stroke (Cheng et al. 2023). Despite this high prevalence, the optimal clinical management of NeP is still challenging mainly due to its complex mechanisms, making both non‐pharmacological and pharmacological approaches necessary (Bouhassira and Attal 2018; Attal and Bouhassira 2021; Zhang et al. 2023). In this setting, gabapentinoids are one of the main classes of drugs used for treatment due to their mechanism of action involving, for example, the interaction with α2δ subunits of voltage‐gated calcium channels (VGCC) (Williams et al. 2023). This VGCC modulation reduces calcium influx into the presynaptic terminal, subsequently decreasing the release of neurotransmitters that act in nociception and amplification of pain signals, such as glutamate, calcitonin gene‐related peptide and substance P (Chincholkar 2018). Moreover, these drugs are suggested to enhance descending inhibition mediated by noradrenaline (Patel and Dickenson 2022; Hayashida and Obata 2019; Hayashida et al. 2007), inhibit synaptic plasticity associated with chronic pain states through interaction with thrombospondin pathways (Yu et al. 2018; Eroglu et al. 2009), and modulate microglial activation, thereby reducing the release of pro‐inflammatory cytokines that correlate with central sensitisation (Wodarski et al. 2009; Scholz and Woolf 2007; Watkins and Maier 2003).

Within this therapeutic framework, mirogabalin has emerged as a novel gabapentinoid, with primary studies suggesting potential efficacy for NeP and pain‐related sleep interference (Kim et al. 2021). Furthermore, translational research suggests that mirogabalin may be less related to central nervous system‐specific adverse events in comparison with older gabapentinoids due to low affinity and rapid dissociation from α2δ‐2 subunits, which are highly expressed in the Purkinje cells (Kim et al. 2021; Domon et al. 2018). However, more evidence is needed to support these claims. Thus, we aimed to perform a systematic review and meta‐analysis of available clinical trials to assess the efficacy and safety of mirogabalin in patients with central or peripheral NeP.

## 2 Material and Methods

We performed and reported this systematic review and meta‐analysis in accordance with the Cochrane Handbook for Systematic Reviews of Interventions (Higgins et al. 2019) and the Preferred Reporting Items for Systematic Reviews and Meta‐Analyses (PRISMA) statements (Page et al. 2021). The protocol was registered prospectively in PROSPERO (Registration ID: CRD 42024546531; registered on May 24, 2024).

Although the initial protocol at its inception included both randomised controlled trials (RCTs) and observational studies, prior to study selection, we restricted the eligibility criteria to RCTs with a placebo comparator. These refinements were implemented to ensure methodological consistency across the included studies and were applied before study selection and data extraction.

The research question and eligibility criteria were structured according to the PICOTT framework:

(P) Patients with neuropathic pain of any severity and aetiology;

(I) Mirogabalin treatment;

(C) Placebo comparator;

(O) Pain intensity (continuous and categorical outcomes), pain‐related sleep interference, clinical global impression, and adverse events;

(T) Randomised controlled trials (type of study);

(T) No restriction on follow‐up duration (time).

### 2.1 Eligibility Criteria

We restricted our analyses to studies that met all the following inclusion criteria: (1) RCTs; (2) comparing mirogabalin with placebo; (3) including patients with NeP of any severity and aetiology and (4) reporting any of the pre‐specified outcomes of interest. There were no eligibility restrictions on population size, follow‐up duration or study language. Studies that did not provide sufficient data for meta‐analysis were excluded.

### 2.2 Search Strategy and Data Extraction

We systematically searched PubMed, Embase, Cochrane Library, Web of Science, and ClinicalTrials.gov from inception up to January 2025. The exact search strategy used can be found in the Supporting Information (Table S1). Two authors (R.B.J and J.O.T) independently screened studies following predefined search criteria and extracted data to a standardised spreadsheet. Disagreements between authors were solved through consensus. In addition, we manually reviewed the references of all included studies and previous relevant reviews (Parker et al. 2019).

### 2.3 Endpoints

The primary outcomes of interest were the mean difference between groups in pain scores extracted from pain diaries and pain‐related sleep interference assessed by diary reports. Additionally, we analysed the mean differences between groups in the patient‐reported pain on the visual analogue scale of the Short‐form McGill Pain Questionnaire (SF‐MPQ) (Melzack 1987), the proportion of patients reporting good (≥ 30%) and substantial (≥ 50%) pain relief, patient‐reported clinical global impression (CGI) scale, and the risk of adverse events.

### 2.4 Quality Assessment

We performed quality assessment with the Cochrane's tool for assessing bias in RCTs [Risk of Bias 2 (RoB2)]. In this tool, the risk of bias is classified as high, some concerns or low in five domains: randomization, deviation from intended interventions, missing outcome data, measurement of the outcome, and selection of the reported result biases (Sterne et al. 2019). Assessment of indirectness was performed based on the applicability of the included studies to the predefined PICOTT framework. No concerns regarding indirectness were identified, as all included studies directly addressed the research question. Publication bias was initially intended to be investigated through analysis of the primary outcome; however, it was not formally assessed because the number of included studies (*n* = 6) was insufficient to generate meaningful results from funnel plot analysis or statistical methods such as Egger's test, as recommended by the Cochrane Handbook (Higgins et al. 2019).

### 2.5 Statistical Analysis

We compared treatment effects for continuous outcomes using the mean difference (MD) between groups and collected absolute numbers for dichotomous outcomes to calculate pooled risk ratios (RRs); estimates were reported with 95% confidence intervals (CI). The generic inverse variance method was used to pool effect sizes. In multi‐arm trials evaluating multiple dosages, results for all doses were combined, unless the combination of means was not possible because of missing data. In this case, only the data for the highest dose was included. We examined the heterogeneity with *I* ^2^ statistics and the Cochran Q test. The DerSimonian and Laird random effects model was used for all outcomes, as recommended by the Cochrane Collaboration (Higgins et al. 2019). For statistical analysis, we used Review Manager Web, version 2024.

## 3 Results

### 3.1 Quality Assessment

The risk of bias appraisal for individual studies is reported in Supplemental Figure 1. One study was considered as overall “low‐risk” of bias in all domains (Ushida et al. 2023). Five studies were considered to have “some concerns” for bias: three due to blinding outcome assessment (Baba et al. 2019, 2020; Merante et al. 2017), one due to attrition bias (Kato et al. 2019), and one due to other bias (Guo et al. 2024). We did not perform a publication bias assessment with funnel‐plot analysis because the number of included studies was insufficient (< 10) (Higgins et al. 2019).

### 3.2 Study Selection and Characteristics

**FIGURE 1:**
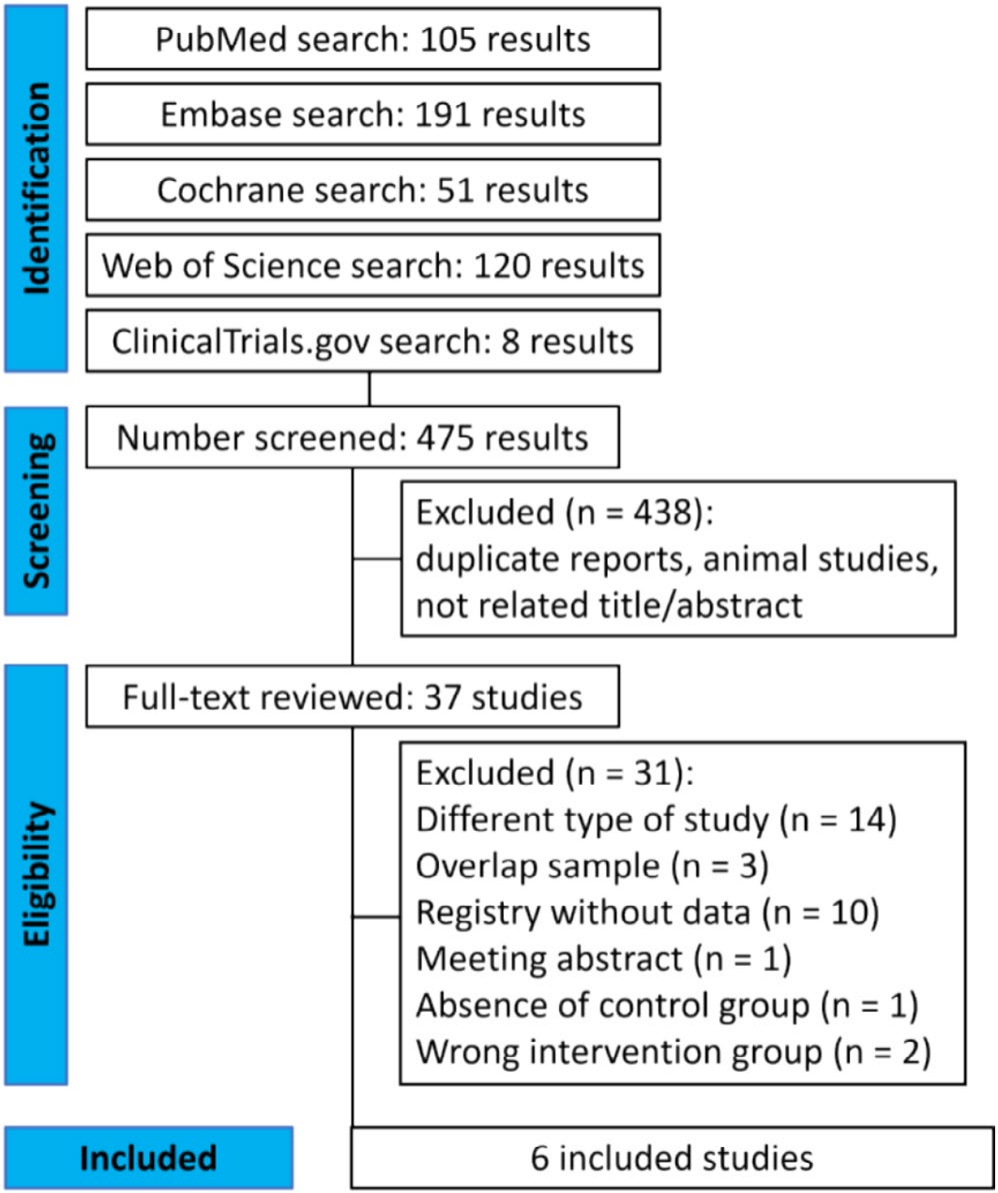
PRISMA flow diagram of study screening and selection.

As shown in Figure 1, we identified 475 results with the search strategy. After the removal of duplicates and excluding non‐relevant studies by title or abstract review, 37 studies were reviewed in full. Of these, six were selected for inclusion, comprising 3048 patients with NeP due to diabetic neuropathy (65.1%), post‐herpetic neuralgia (25.1%), or spinal cord injury (9.8%) from double‐blind parallel RCTs (Baba et al. 2019, 2020; Kato et al. 2019; Merante et al. 2017; Ushida et al. 2023; Guo et al. 2024). No additional studies were identified for inclusion based on the forward/backward citation tracking method.

The mean age of participants was 60.6 years, and 64.7% were male. The duration of treatment ranged from 5 to 14 weeks. Additional baseline characteristics of these studies are shown in Table 1.

**TABLE 1:** Baseline characteristics of the included studies (randomised controlled trials).

Study, year	Location	Sample (MGB/Placebo)	Mean age, years	Male (%)	Clinical condition associated with NeP	Daily dose range	Treatment duration (weeks)
Baba et al. 2019	Multicentric	500/334	61.4	72.5	Diabetic neuropathy	15–30 mg	14
Baba et al. 2020	Multicentric	273/88	50.7	63.2	Diabetic neuropathy	10–30 mg	7
Kato et al. 2019	Multicentric	304/461	66.5	60.3	Postherpetic neuralgia	15–30 mg	14
Merante et al. 2017	Multicentric	284/112	60.2	53	Diabetic neuropathy	5–30 mg	5
Ushida et al. 2023	Multicentric	150/149	58.5	85.6	Spinal cord injury	10–30 mg	14
Guo et al. 2024	Multicentric	196/197	58.2	54.2	Diabetic neuropathy	10–30 mg	14

Abbreviations: MGB, mirogabalin; NeP, neuropathic pain.

### 3.3 Pooled Analysis of All Studies

**FIGURE 2:**
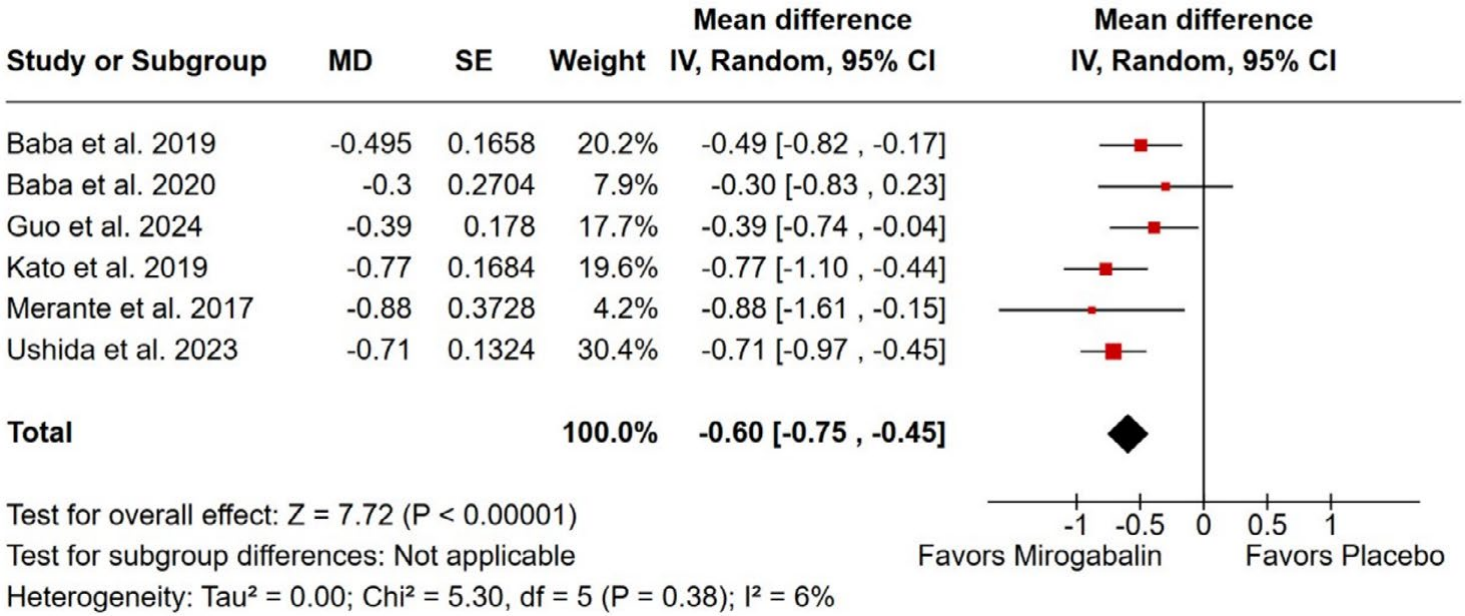
There was a significant decrease in pain intensity (as measured by average daily pain score) in patients affected by neuropathic pain treated with mirogabalin as compared with placebo.

**FIGURE 3:**
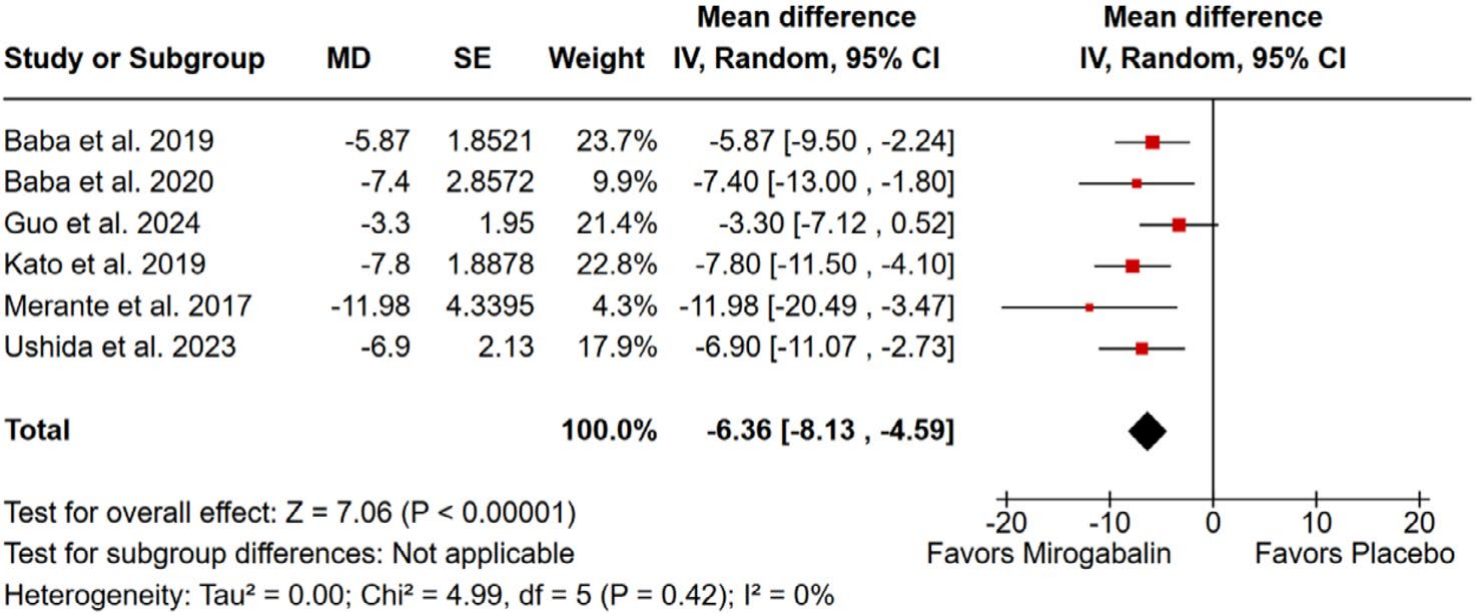
There was a significant decrease in patient‐reported pain, as measured by the visual analogue scale of the Short‐form McGill Pain Questionnaire, in patients affected by neuropathic pain treated with mirogabalin as compared with placebo.

Pain intensity decreased in the group treated with mirogabalin compared with placebo, as measured by average daily pain scores ranging from 0 to 10 (MD −0.60; 95% CI −0.75 to −0.45; *p* < 0.001; *I* ^2^ = 6%; Figure 2) and by the visual analogue scale of the SF‐MPQ (MD −6.36; 95% CI −8.13 to −4.59; *p* < 0.001; *I* ^2^ = 0%; Figure 3).

**FIGURE 4:**
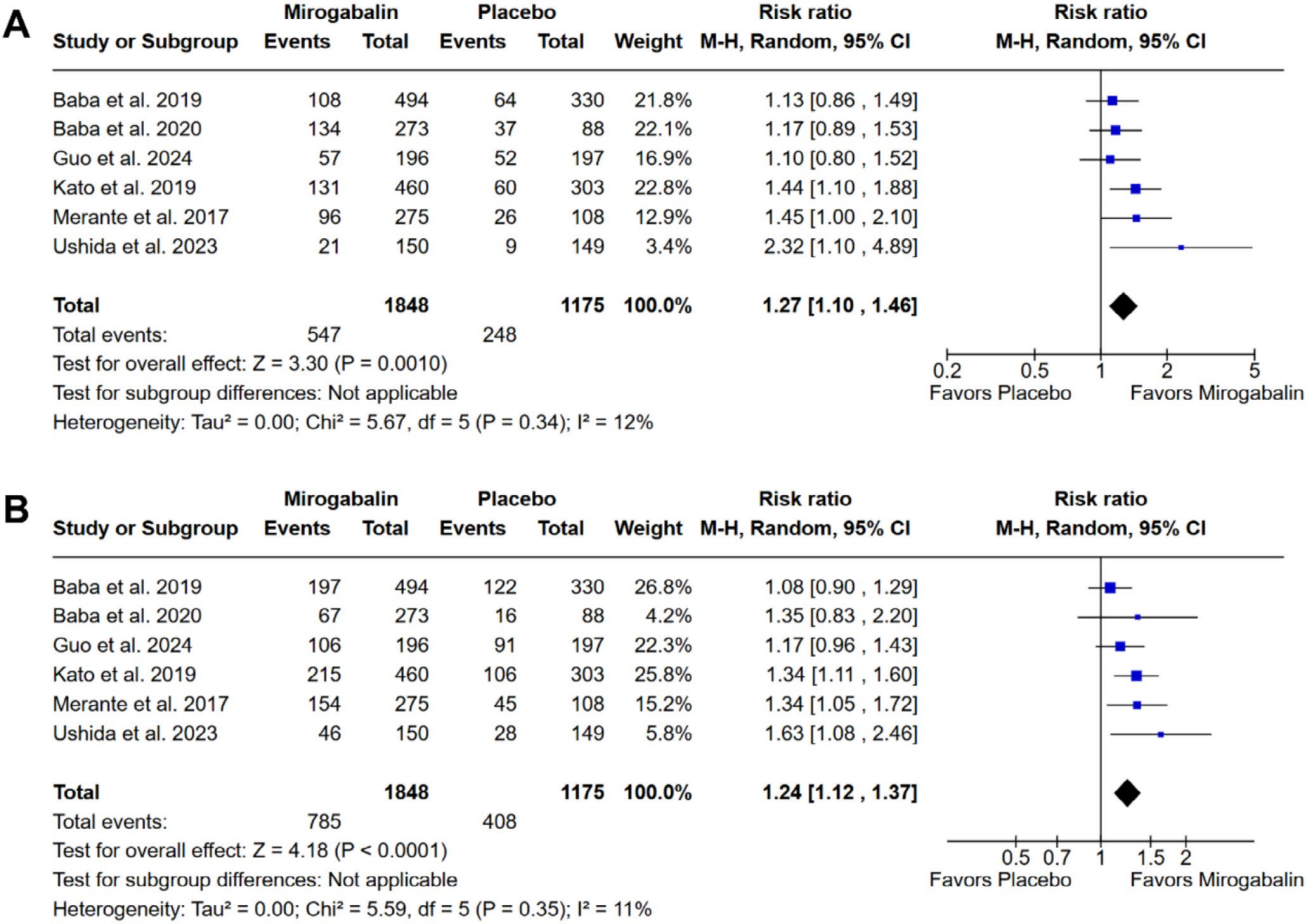
There was a higher proportion of patients with neuropathic pain intensity relief ≥ 50% (A) and ≥ 30% (B) in the group treated with mirogabalin as compared with placebo.

There was a higher proportion of patients reporting pain relief ≥ 50% (RR 1.27; 95% CI 1.10 to 1.46; *p* = 0.001; *I* ^2^ = 12%; Figure 4A), as well as ≥ 30% (RR 1.24; 95% CI 1.12 to 1.37; *p* < 0.001; *I* ^2^ = 11%; Figure 4B) in the group treated with mirogabalin versus placebo.

**FIGURE 5:**
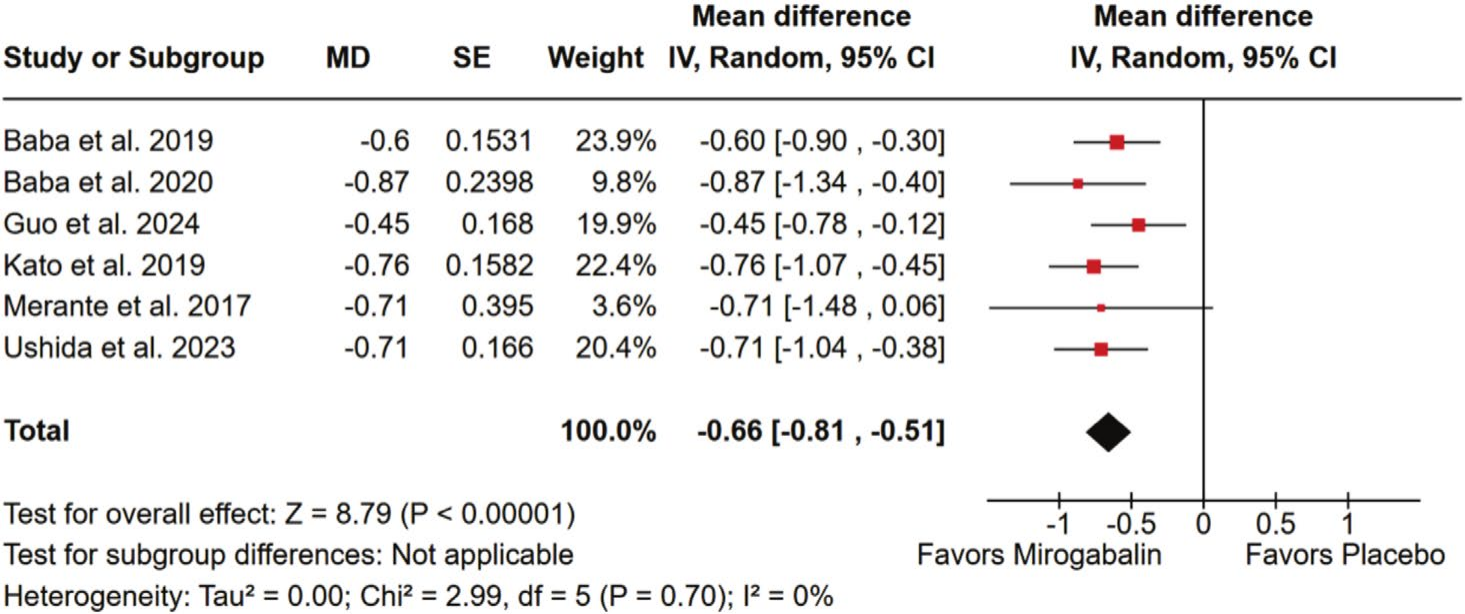
There was a significant improvement in pain‐related sleep interference in patients affected by neuropathic pain treated with mirogabalin compared with placebo.

There was a significant improvement in pain‐related sleep interference, as measured on a 10‐point numerical rating scale, in patients treated with mirogabalin relative to placebo (MD −0.66; 95% CI −0.81 to −0.51; *p* < 0.001; I^2^ = 0%; Figure 5).

**FIGURE 6:**
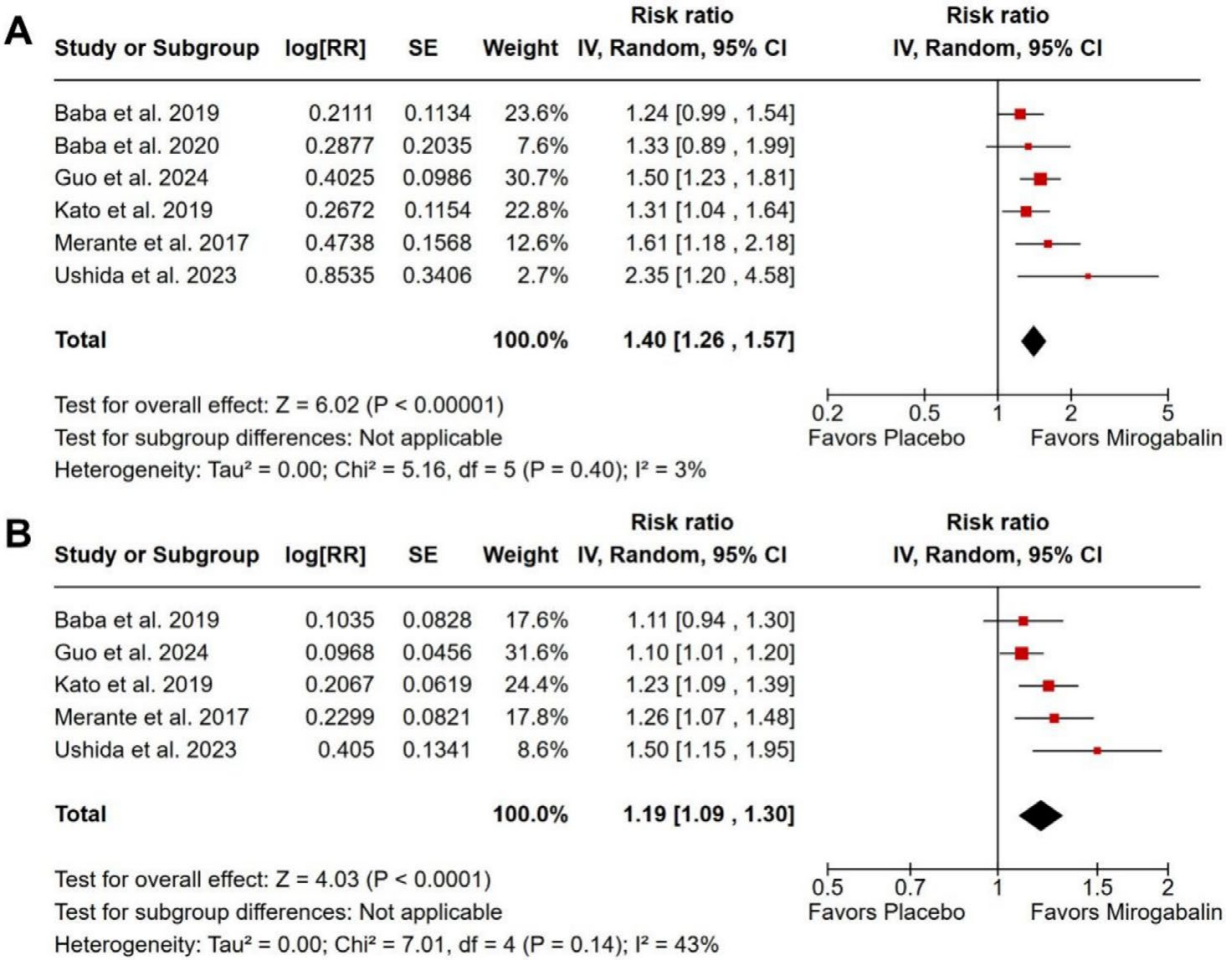
There was a higher proportion of patients with NeP reporting “much improved or very much improved” (A) or at least some perceived improvement (B) on the patient‐reported clinical global impression scale in the group treated with mirogabalin compared with placebo.

A higher proportion of patients reported “much improved or very much improved” on the patient‐reported CGI scale (RR 1.40; 95% CI 1.26 to 1.57; *p* < 0.001; *I* ^2^ = 3%; Figure 6A), or at least some perceived improvement on the same assessment in the mirogabalin group relative to placebo (RR 1.19; 95% CI 1.09 to 1.30; *p* < 0.001; *I* ^2^ = 43%; Figure 6B). The assessment of heterogeneity across outcomes showed predominantly low values. The heterogeneity for the primary outcome (pain intensity reduction) was low (*I* ^2^ = 6%). Heterogeneity was also low across most secondary outcomes (*I* ^2^ ranging from 0% to 12%), except for the outcome “at least some perceived improvement”, which showed moderate heterogeneity (*I* ^2^ = 43%; Figure 6B).

Compared with placebo, treatment with mirogabalin was associated with a higher risk of adverse events, including weight gain (RR 4.86; 95% CI 2.44 to 9.69; *p* < 0.001), peripheral oedema (RR 4.42; 95% CI 2.46 to 7.92; *p* < 0.001), somnolence (RR 3.68; 95% CI 2.67 to 5.08; *p* < 0.001), dizziness (RR 2.86; 95% CI 1.98 to 4.15; *p* < 0.001), and nasopharyngitis (RR 1.34; 95% CI 1.05 to 1.70; *p* = 0.02; Table 2).

**TABLE 2:** Pooled adverse events of mirogabalin compared to placebo in patients with neuropathic pain.

Adverse event	Mirogabalin (events/total)	Placebo (events/total)	Pooled RR [95% CI]	*p* value†	I^2^
Weight gain	78/1851	9/1174	4.86 [2.44–9.69]	< 0.001	0%
Peripheral oedema	90/1851	13/1174	4.42 [2.46–7.92]	< 0.001	0%
Somnolence	255/1851	44/1174	3.68 [2.67–5.08]	< 0.001	3%
Dizziness	162/1851	34/1174	2.86 [1.98–4.15]	< 0.001	0%
Nasopharyngitis	167/1378	79/869	1.34 [1.05–1.70]	0.02	0%

Abbreviations: I^2^, Heterogeneity Index; RR, Risk Ratio.

^†^ *p* value for test of overall effect.

### 3.4 Sensitivity Analysis

We performed leave‐one‐out analyses to confirm that any included studies did not significantly impact our main results. Changes in average daily pain scores, visual analogue scale scores, and average daily sleep interference scores remained statistically significant (and with low heterogeneity) after the individual exclusion of every study (Figures S2–S4). Moreover, considering the variability in the follow‐up duration across the included studies, we conducted additional analyses focusing exclusively on the four trials with a 14‐week follow‐up duration (Baba et al. 2019; Kato et al. 2019; Ushida et al. 2023; Guo et al. 2024). This implied the exclusion of two trials with five and seven weeks of duration (Merante et al. 2017; Baba et al. 2020). No significant changes were observed in the pooled results of the scores mentioned above (Figure S5).

## 4 Discussion

Our meta‐analysis of RCTs evaluated the efficacy and safety of mirogabalin in the treatment of patients with NeP secondary to diabetic neuropathy, post‐herpetic neuralgia, or spinal cord injury. The main findings were as follows: (1) mirogabalin significantly improved pain outcomes in comparison with placebo, measured both as a continuous variable or as a binary outcome of pain improvement of ≥ 50% or ≥ 30% from baseline; (2) similarly, the interference of NeP on sleep was decreased in patients treated with mirogabalin and (3) there was a higher incidence of adverse events such as weight gain, somnolence, peripheral oedema, and dizziness as compared with placebo.

Although the findings of this meta‐analysis were statistically significant across primary and secondary outcomes, the magnitude of the observed effects was modest. The mean difference in pain reduction (MD −0.60) and in pain‐related sleep interference (MD −0.66) suggests that, while mirogabalin provided a statistical benefit compared with placebo, the clinical improvements were relatively small. These results align with previous reports on gabapentinoids for NeP, which show that statistically significant benefits may not necessarily correlate with robust clinical gains. For example, the number needed to treat (NNT) for gabapentinoids in NeP ranges from approximately 4 to 8 in conditions such as post‐herpetic neuralgia, diabetic neuropathy, and central neuropathic pain; meta‐analyses report combined NNTs of approximately 7.2 for gabapentin and 7.7 for pregabalin (Moore et al. 2014; Wiffen et al. 2017; Derry et al. 2019; Finnerup et al. 2015). Nonetheless, in terms of personalised care, even modest changes can improve the overall quality of life for specific individuals (O'Connor 2009). From a translational perspective, advances in pharmacogenomics may enable more tailored use of gabapentinoids—including mirogabalin—by identifying individuals most likely to experience meaningful therapeutic benefits (Mackenzie et al. 2024; Shaheen et al. 2021).

The novel gabapentinoid mirogabalin is a selective α2δ‐1 and α2δ‐2 ligand with slower dissociation from α2δ‐1 and faster unbinding from α2δ‐2 subunits of VGCC. Such a feature could contribute to stronger analgesic efficacy and to a theoretically safer adverse events profile in comparison with gabapentin or pregabalin (Zajączkowska et al. 2021; Kim et al. 2021; Domon et al. 2018). However, clinical data so far have not consistently demonstrated a significantly improved tolerability profile compared to existing gabapentinoids. A meta‐analysis of RCTs assessing patients with NeP due to diabetic neuropathy over seven weeks showed that mirogabalin was superior not only to placebo but also to pregabalin (300 mg/day) in decreasing average daily pain and average daily sleep interference by pain scores. Although mirogabalin was associated with higher incidences of adverse events (e.g., dizziness, weight gain, peripheral oedema, and somnolence) compared with placebo, its safety profile was similar to pregabalin. However, this pooled outcome analysis included only two RCTs comparing mirogabalin and pregabalin (Alyoubi et al. 2021), making further studies, such as head‐to‐head trials, necessary.

Mirogabalin was shown to achieve its maximum plasma concentration in approximately 0.5 to 1 h, compared to around 1 h for pregabalin and 2 to 3 h for gabapentin (Kim et al. 2021). Additionally, mirogabalin's dosing regimen differs from that of gabapentin and pregabalin. While conventional gabapentin is required to be taken three to four times a day, mirogabalin may be taken twice daily. Compared to pregabalin (which can also be dosed twice a day), mirogabalin has been associated with more stable serum levels and longer duration of action (Kim et al. 2021). However, the clinical relevance of these pharmacokinetic differences remains to be fully established. A previous study showed that mirogabalin 30 mg/day was associated with a frequency of 7% of adverse effects leading to discontinuation, compared with 18%–28% for pregabalin 600 mg/day, 12% for gabapentin ≥ 1200 mg/day, and 12.5% for duloxetine (Javed et al. 2018). On the other hand, mirogabalin, similarly to other gabapentinoids, needs to have its dose adjusted for patients with moderate to severe renal dysfunction, which partially limits its use in this population (Yin et al. 2016).

Gabapentinoids do not address all the mechanisms underpinning NeP (Attal and Bouhassira 2015; Bouhassira and Attal 2019). Other treatment options include tricyclic and dual antidepressants (Rosner et al. 2023), opioids (Fung and Kang 2023), cannabinoids (Rog et al. 2005; Meng et al. 2017), physiotherapy (Kannan et al. 2022), acupuncture (Ma et al. 2022), and neuromodulation techniques (Galafassi et al. 2021), each with varying degrees of efficacy and specific limitations. Thus, although new drugs are becoming available, the growth of precision medicine remains critical for the management of NeP, targeting individuals' characteristics, genetic markers, and pain mechanisms (Bouhassira and Attal 2023; Calvo et al. 2019).

We synthesised evidence from six placebo‐controlled RCTs assessing mirogabalin for NeP and pain‐related sleep interference (Baba et al. 2019, 2020; Kato et al. 2019; Merante et al. 2017; Ushida et al. 2023; Guo et al. 2024), demonstrating efficacy across both outcomes. Notwithstanding the therapeutic benefits, this meta‐analysis revealed an increased incidence of adverse events related to mirogabalin compared with placebo, including weight gain, peripheral oedema, somnolence, and dizziness. These findings align with results commonly reported in the literature for gabapentinoids (Chincholkar 2018; Benarroch 2021). Despite mirogabalin's pharmacokinetic advantages and greater selectivity for the α2δ‐1 subunit, its overall adverse event profile remains within the expected therapeutic range for this drug class (Benarroch 2021; Zajączkowska et al. 2021). Thus, proactive management strategies, including dose titration and patient education, are paramount to mitigate adverse events and optimise favourable outcomes (Alles et al. 2017).

Although our findings provide updated evidence on efficacy and tolerability, further investigation is warranted to address remaining knowledge gaps. For instance, a recent large‐scale case–case‐time‐control study in Japan found that mirogabalin use was associated with an increased risk of fracture (OR 1.53; 95% CI 1.35–1.72), similar to pregabalin (OR 1.35; 95% CI 1.28–1.43), particularly among older adults (Wakabayashi et al. 2025). Additional high‐quality RCTs—including head‐to‐head comparisons with other gabapentinoids—and long‐term evaluations of efficacy, safety, and cost‐effectiveness are necessary to characterise the clinical profile of mirogabalin across diverse NeP syndromes.

Notably, growing evidence supports mirogabalin's efficacy and tolerability. Although previous international guidelines did not incorporate this agent (Andrejic et al. 2025; Moisset et al. 2021, 2020; Finnerup et al. 2021; Attal et al. 2010; Schlereth 2020), the updated NeuPSIG recommendations have recently included mirogabalin as a first‐line pharmacologic option for treating NeP (Soliman et al. 2025). This aligns with its already established use in clinical practice in several Asian countries (Baba et al. 2019; Guo et al. 2024; Kato et al. 2019; Suzuki et al. 2024; Obara et al. 2024; Fujii et al. 2024; Tadokoro et al. 2024).

Certain additional limitations were noteworthy. First, the small number of included studies precluded additional analyses, such as additional subgroup evaluation and publication bias assessment. Second, we were not able to assess long‐term outcomes, as most of the included studies only reported results within controlled phases. Third, there was a heterogeneity of NeP syndromes among the included clinical trials, which makes further studies with larger and more homogeneous populations necessary. In addition, Daiichi Sankyo Inc. sponsored all RCTs included in this meta‐analysis, and several authors across the studies reported affiliations with the company. While all trials employed rigorous randomised, double‐blinded designs, it is recognised that sponsorship may be an inherent source of influence in clinical research. This factor should be considered when interpreting the overall findings.

Finally, we should acknowledge a potential limitation of the review process. While our strategy encompassed major databases and trial registries, other sources of grey literature—such as conference abstracts, dissertations, and regulatory reports—were not systematically explored. However, we believe that the comprehensive search across five major databases, together with manual reference screening and the inclusion of registry data, was sufficient to capture the relevant and methodologically sound evidence available to date.

## 5 Conclusion

In this meta‐analysis of RCTs assessing patients with NeP, mirogabalin was associated with a significant improvement in pain and pain‐related sleep interference as compared with placebo; however, there was an increased risk of adverse events. Further head‐to‐head trials and long‐term studies are warranted to define its optimal use and support its inclusion in broader clinical guidelines.

## Author Contributions

**Rafael Batista João:** conceptualisation, data curation, formal analysis, investigation, validation, project administration, supervision, visualisation, writing – original draft, writing – review and editing. **Jilly Octoria Tagore Chan:** data curation, formal analysis, investigation, validation, visualisation, writing – original draft, writing – review and editing. **André Batista João:** data curation, formal analysis, investigation, validation, visualisation, writing – original draft, writing – review and editing. **Luísa Mendes Araújo:** investigation, validation, visualisation, writing – original draft, writing – review and editing. **Julyana Medeiros Dantas:** conceptualisation, formal analysis, investigation, validation, project administration, supervision, writing – review and editing.

## Disclosure

The authors have nothing to report.

## Ethics Statement

The authors have nothing to report.

## Consent

All authors have approved and consented to the publication of this paper.

## Conflicts of Interest

The authors declare no conflicts of interest.

## Supporting information


**Data S1:** ejp70112‐sup‐0001‐Supinfo.docx.

## Data Availability

The authors have nothing to report.
