# Supplementary material for: Mirogabalin for Treatment of Neuropathic Pain and Associated Sleep Interference: An Updated Meta‐Analysis
Source: Eur J Pain. 2025 Sep 19;29(10):e70112. doi: 10.1002/ejp.70112 (PMC12447546; doi:10.1002/ejp.70112)
Supplement: Supplementary file 1 — Data S1: ejp70112‐sup‐0001‐Supinfo.docx. [file EJP-29-0-s001.docx]

***Supplementary material***

| **Supplemental Table 1.** Complete search strategy. | |
| --- | --- |
| PubMed, Embase, Cochrane Library, Web of Science, and ClinicalTrial.Gov | (Mirogabalin OR DS-5565 OR Tarlige) AND  ("Neuropathic Pain” OR neuropathy OR neuropathic) |


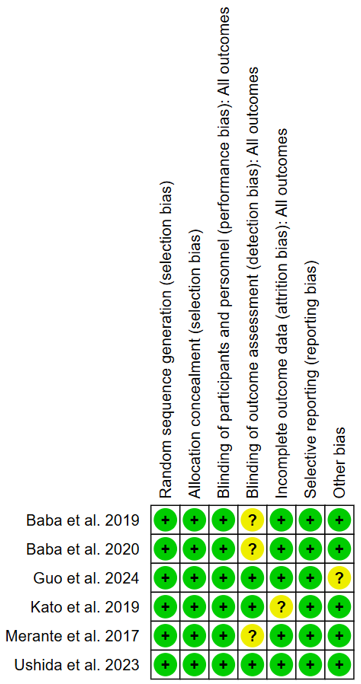


**Supplemental Figure 1 – Risk of Bias (RoB2) assessment.**


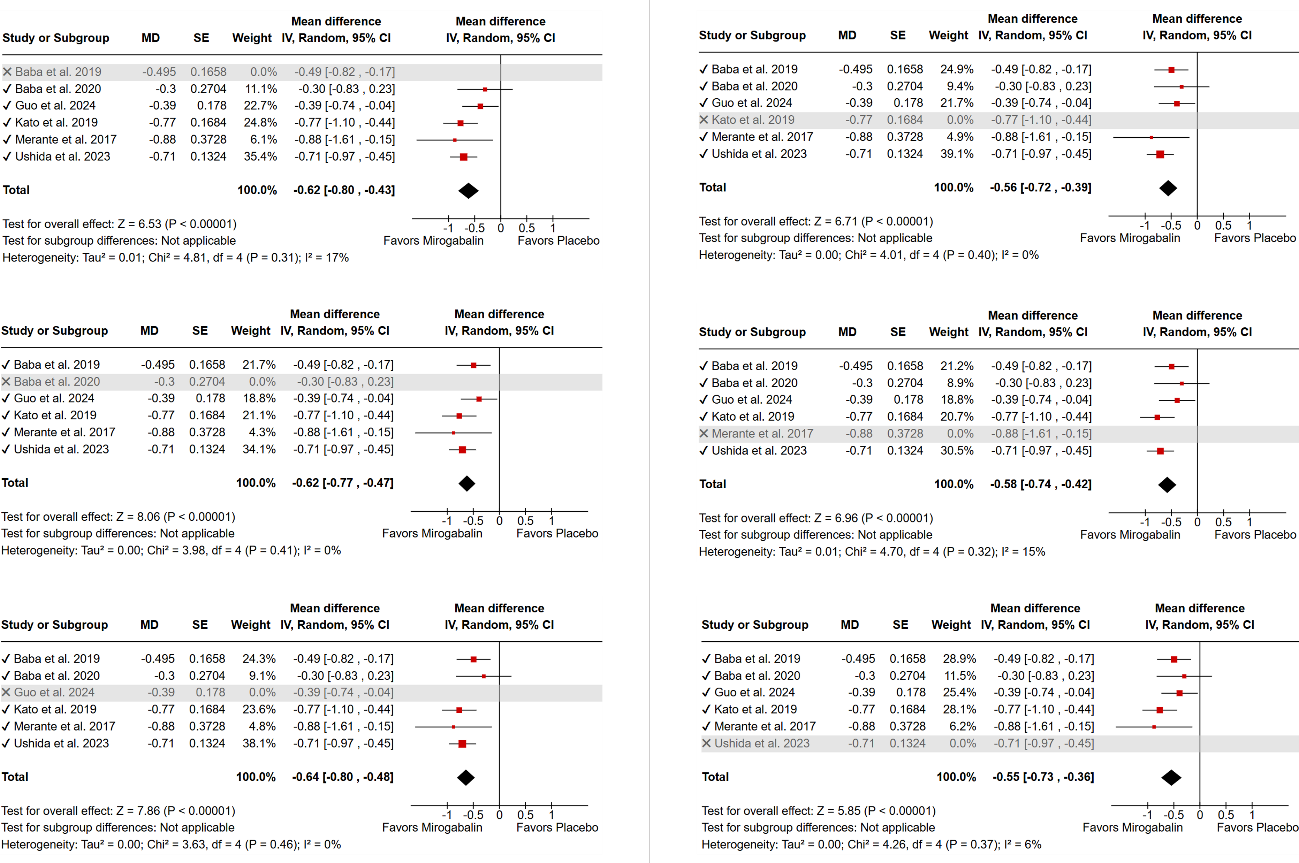


**Supplemental Figure 2 – The leave-one-out sensitivity analysis did not show significant differences in the overall pooled results of pain scores as assessed by ADPS.**

**
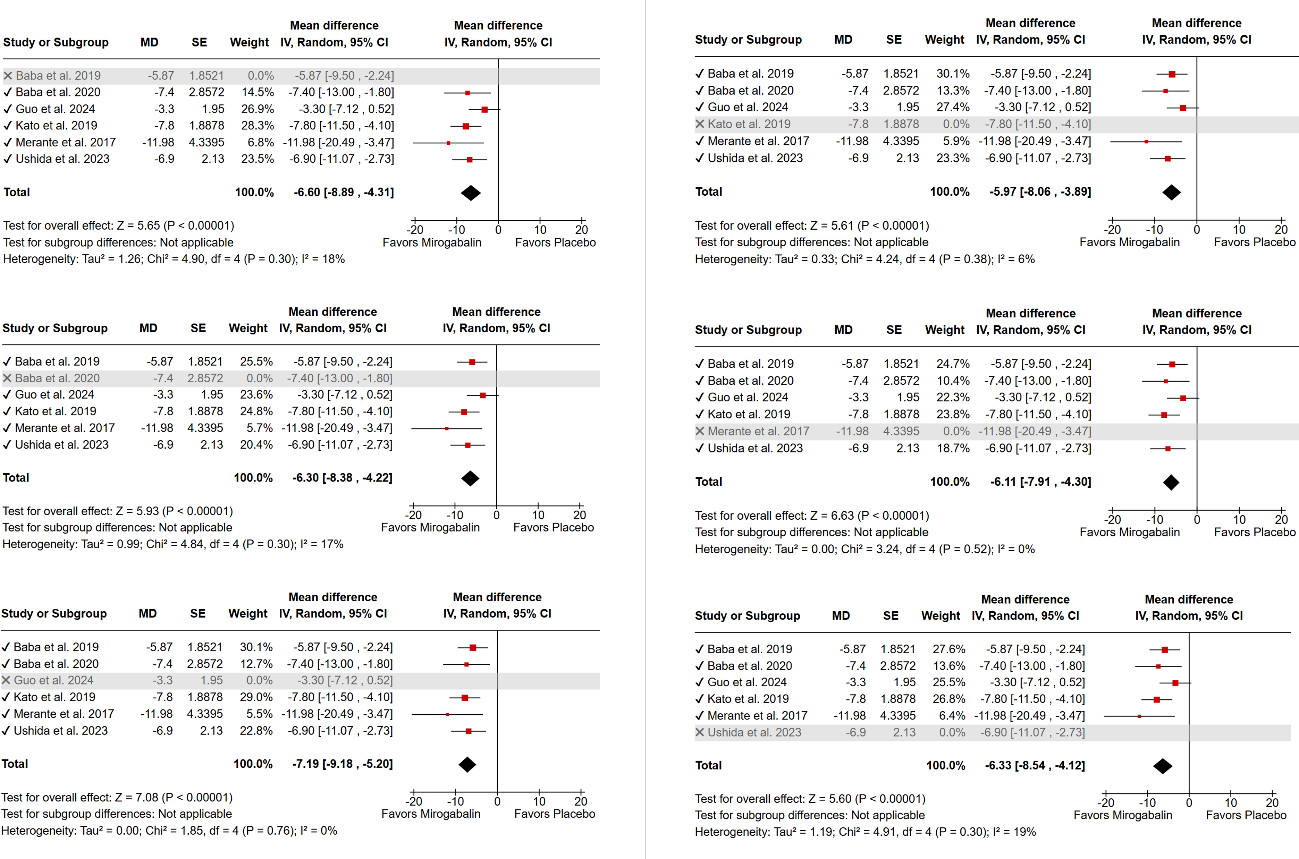
**

**Supplemental Figure 3 – The leave-one-out sensitivity analysis did not show significant differences in the overall pooled results of pain scores as assessed by VAS.**

**
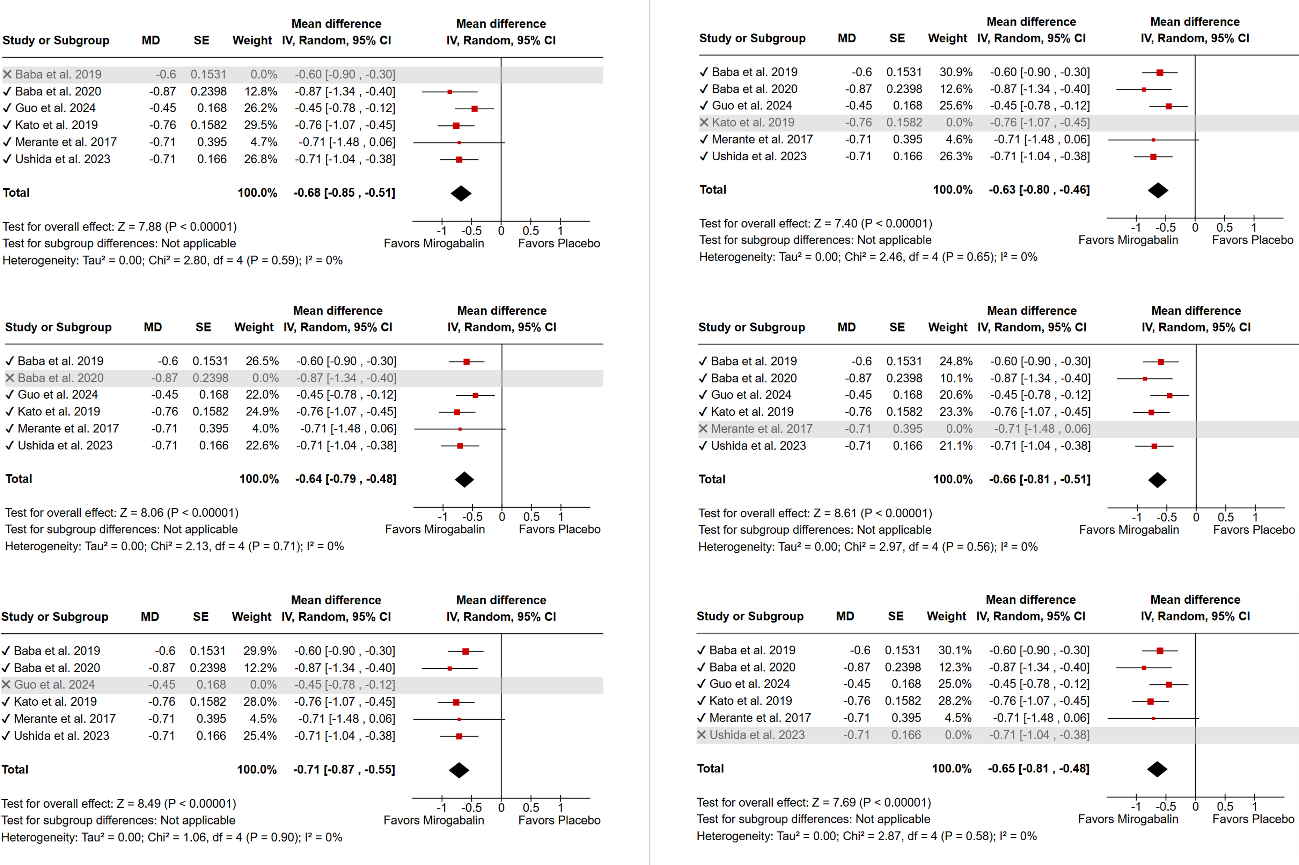
**

**Supplemental Figure 4 – The leave-one-out sensitivity analysis did not show significant differences in the pooled pain sleep interference scores assessed by ADSIS.**

**
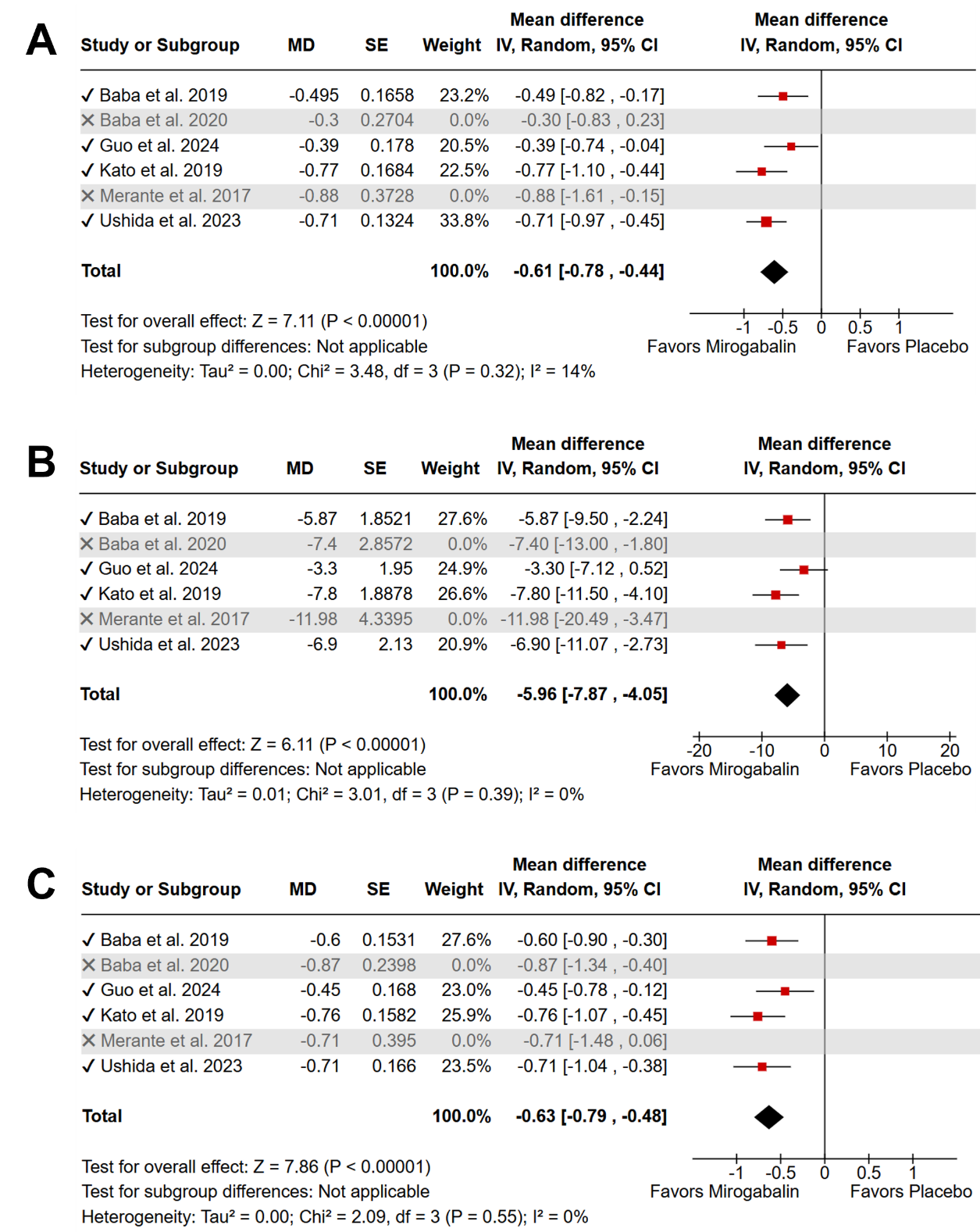
**

**Supplemental Figure 5 – The sensitivity based on the exclusion of the two trials with less than 14 weeks of follow-up duration (e.g., 5- and 7-weeks duration) did not show significant differences in the overall pooled results of ADPS (A), VAS (B), and ADSIS (C).**
